# Transcription factor activating protein 4 is synthetically lethal and a master regulator of *MYCN-*amplified neuroblastoma

**DOI:** 10.1038/s41388-018-0326-9

**Published:** 2018-06-07

**Authors:** Shuobo Boboila, Gonzalo Lopez, Jiyang Yu, Debarshi Banerjee, Angela Kadenhe-Chiweshe, Eileen P. Connolly, Jessica J. Kandel, Presha Rajbhandari, Jose M. Silva, Andrea Califano, Darrell J. Yamashiro

**Affiliations:** 1 Department of Pediatrics, Columbia University Medical Center, New York, NY 10032 USA; 2 Department of Pathology and Cell Biology, Columbia University Medical Center, New York, NY 10032 USA; 3 Department of Radiation Oncology, Columbia University Medical Center, New York, NY 10032 USA; 4 Department of Systems Biology, Columbia University Medical Center, New York, NY 10032 USA; 5 Department of Pediatrics, Perelman School of Medicine, University of Pennsylvania, Philadelphia, PA 19104 USA; 6 Department of Surgery, Columbia University Medical Center, New York, NY 10032 USA; 7 Department of Surgery, Section of Pediatric Surgery, University of Chicago Medicine & Biological Sciences, Chicago, IL 60637 USA; 8 Department of Biological Sciences, Columbia University, New York, NY 10025 USA; 9 Department of Pathology, Icahn School of Medicine at Mount Sinai, New York, NY 10029 USA; 10 Present Address: Division of Oncology and Center for Childhood Cancer Research, Children’s Hospital of Philadelphia, Philadelphia, PA 19104 USA; 11 Present Address: Department of Computational Biology, St. Jude Children’s Research Hospital Kay Research and Care Center, IA6053, 262 Danny Thomas Place, Mail Stop 1135, Memphis, TN 38105-3678 USA

## Abstract

Despite the identification of *MYCN* amplification as an adverse prognostic marker in neuroblastoma, MYCN inhibitors have yet to be developed. Here, by integrating evidence from a whole-genome shRNA library screen and the computational inference of master regulator proteins, we identify transcription factor activating protein 4 (TFAP4) as a critical effector of *MYCN* amplification in neuroblastoma, providing a novel synthetic lethal target. We demonstrate that TFAP4 is a direct target of *MYCN* in neuroblastoma cells, and that its expression and activity strongly negatively correlate with neuroblastoma patient survival. Silencing TFAP4 selectively inhibits *MYCN-*amplified neuroblastoma cell growth both in vitro and in vivo, in xenograft mouse models. Mechanistically, silencing TFAP4 induces neuroblastoma differentiation, as evidenced by increased neurite outgrowth and upregulation of neuronal markers. Taken together, our results demonstrate that TFAP4 is a key regulator of *MYCN*-amplified neuroblastoma and may represent a valuable novel therapeutic target.

## Introduction

Neuroblastoma is the most common extracranial solid tumor in childhood, accounting for 13% of all pediatric cancer mortality. Overall survival of high-risk patients remains < 50%, despite intensive therapy with high-dose chemotherapy, surgery, stem cell transplantation, and immunotherapy [1]. *MYCN* amplifications represent one of the major genetic alterations that correlates with poor prognosis. *MYCN* was first identified in neuroblastoma as a *c-myc*-related oncogene [2, 3], and is amplified in ~25% of neuroblastoma cases [4]. Despite the knowledge that *MYCN* amplifications represent an adverse prognostic marker, no FDA-approved or late-stage investigational compound has been developed to target MYCN directly.

An alternative approach to treating *MYCN-*amplified neuroblastoma patients would be to target proteins whose essentiality is only manifested in *MYCN-*amplified tumors (*MYCN*^Amp^ synthetic lethal). Several synthetic lethal screens with *MYC/MYCN* overexpression have been reported across different cancers [5–8], leading to identification of the bromodomain protein, BRD4, as a common hit in these screens. The small molecule BRD4 inhibitor JQ1 has been shown to downregulate the MYC transcriptional network and inhibit tumor growth of MYC-driven multiple myeloma [9], lymphoma [10], and neuroblastoma [11]. This suggests that synthetic lethal screens can successfully identify novel drug targets for cancers.

A complementary approach to identifying novel drug targets is through computational analysis of gene regulatory networks [12]. Specifically, the Master Regulator Inference Algorithm (MARINa) was developed to identify aberrantly activated/inactivated *Master Regulator* (MR) proteins, representing tumor drivers [13, 14]. Subsequently, the virtual inference of protein activity by enriched regulon analysis (VIPER) transforms the gene expression profile of samples into a protein activity profiles [15]. The MARINa and VIPER analysis have helped elucidate novel mechanisms of tumorigenesis, progression and drug sensitivity in glioma [13], leukemia [16], lymphoma [17], prostate [18], breast cancer [19], and recently in neuroblastoma [20].

Following the reasoning in our earlier study on glucocorticoid resistance in T-ALL [16], we hypothesized that synthetic lethal genes would be identified as MRs that mechanistically regulate the transcriptional signature associated with *MYCN-*amplified neuroblastoma. Thus, we combined evidence from VIPER-based predictions of *MYCN*-amplified neuroblastoma MRs with a whole-genome shRNA screen. This analysis identified transcriptional factor activating protein 4 (TFAP4) as the top synthetic lethal candidate and *MYCN*-amplified neuroblastoma subtype MR. Mechanistically, we show that TFAP4 is a direct downstream transcriptional target and key effector of MYCN activity, and that silencing it inhibits *MYCN-*amplified cell growth both *in vitro* and *in vivo*. We demonstrate that TFAP4 functions to promote proliferation and inhibit differentiation in *MYCN-*amplified neuroblastoma.

## Results

### TFAP4 is a synthetic lethal candidate and master regulator of *MYCN-*amplified neuroblastoma

**Fig. 1:**
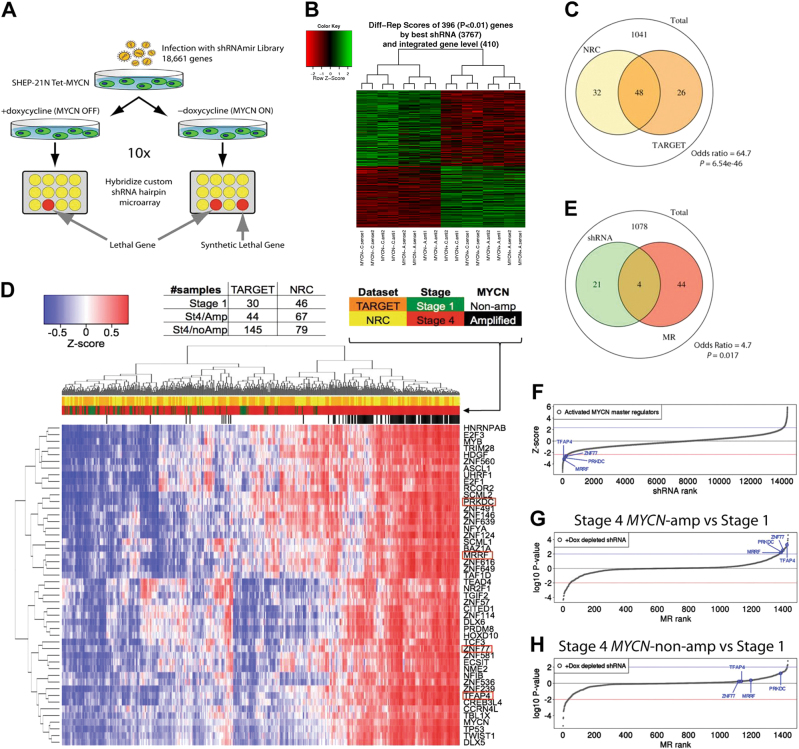
TFAP4 is a synthetic lethal candidate and master regulator of *MYCN-*amplified neuroblastoma. **a** Schema of whole-genome shRNA screening in SHEP21N neuroblastoma cells. Abundance of an individual shRNA was determined by hybridization to a customized microarray. Synthetic lethal candidates are the shRNAs depleted in MYCN ON population only; **b** Heatmap of differentially expressed genes in MYCN ON vs. MYCN OFF conditions. Abundance of shRNAs against 396 genes significantly changed (*P* < 0.01), with 218 genes depleted in the MYCN ON population. **c** Venn diagram of overlapping MRs activated in *MYCN*-amplified tumors (*P* < 0.01), between NRC (yellow circle) and TARGET (orange circle) cohorts. There are 1041 genes tested in both data sets. **d** VIPER single sample activity heatmap for 48 MRs of *MYCN*-amplified neuroblastoma from TARGET and NRC intersected results. Blue–red colors indicate *z*-score scaled enrichment scores from single sample analysis. The four MRs that are synthetic lethal with MYCN are highlighted in red rectangles. **e** Venn diagram of overlapping transcriptional regulators in the synthetic lethal screen and MRs in *MYCN-*amplified neuroblastoma. Twenty-five transcriptional regulators that were significantly depleted in the shRNA screen (green circle) and 48 activated MRs identified by MARINA algorithm (red circle). There are 1078 genes tested in both data sets. **f** Four candidate activated MYCN MRs (*TFAP4, MRRF, PRKDC, ZNF77*), were depleted in the shRNA screen. shRNA differential change was ranked by *z*-score with significantly depleted shRNAs having a z-score of < −2.326 (one-tiled *P* < 0,01) (red line). **g** 1344 transcriptional regulators were ranked by the significance of differential expression of its regulon in *MYCN-*amplified compared with Stage 1. The curve represents the distribution with the most repressed MRs at left tail end and the most activated MRs at right tail end. The candidate MYCN MRs (*TFAP4, MRRF, PRKDC, ZNF77*), were significantly activated (log10 *P* value, > 2, blue line). **h** 1344 transcriptional regulators were ranked by the significance of differential expression of its regulon in non-*MYCN*-amplified stage 4 compared with Stage 1. None of the candidate MYCN MRs (*TFAP4, MRRF, PRKDC, ZNF77*) were significantly activated (log10 *P* value, > 2, blue line)

As a first step to identify novel synthetic lethal candidates to *MYCN* amplification, we performed a whole-genome shRNA screen (Fig. 1a). A pooled shRNA lentiviral library consisting 58,493 shRNA-mirs targeting 18,661 known human genes [21], was used to infect the neuroblastoma cell line SHEP-21 N [22]. SHEP-21 N is a MYCN single copy neuroblastoma cell line that expresses high levels of a *MYCN* transgene (Supplementary Fig. S1). *MYCN* expression can be switched off by adding tetracycline/doxycycline (Tet-off system) *MYCN* expression was decreased by > 90%.

SHEP-21 N cells infected with the shRNA-mir library were puromycin selected and split evenly into two populations: one without doxycycline (MYCN ON) and the other one with doxycycline (MYCN OFF). Total genomic DNA of these two populations was collected after ten cell doubling times, and the shRNA region was PCR amplified. We identified shRNA candidates using a customized microarray and statistical analysis methods as previously described [23]. We identified 396 shRNAs that were differentially expressed between the two populations (*P* < 0.01), of which 218 were significantly depleted in the MYCN ON population, and were therefore *MYCN* synthetic lethal candidates (Fig. 1b, Supplementary Table 1).

We then proceeded to assess whether any of the genes identified by the pooled screen analysis could also be validated as MRs in *MYCN*-amplified patients. VIPER [15] was used to interrogate two neuroblastoma patient data sets: (1) the National Cancer Institute (NCI) Therapeutically applicable research to generate effective treatments (TARGET) data set, comprising 249 gene expression profiles from primary tumor samples; and (2) the European Neuroblastoma Research Consortium (NRC) data set, comprising an additional 283 profiles. Two independent transcriptional interaction networks (interactomes) were assembled by ARACNe analysis of each data set. We calculated differential expression signatures between *MYCN*-amplified tumors (TARGET *n* = 44; NRC *n* = 67) and Stage 1 tumors (TARGET *n* = 30; NRC *n* = 46). Proteins that were identified as statistically significantly aberrantly activated (*P* < 0.01) in both analyses were selected as high-confidence candidate MRs. Overall, there were 74 aberrantly activated MRs from the TARGET analysis and 80 from the NRC. There was a remarkable concordance between the two independent analyses, with 48 activated MRs in common (Odds ratio = 64.7; Fisher’s exact test, *P* = 6.56e-46, Fig. 1c). Differential activity of these 48 MRs in *MYCN*-amplified patients compared with Stage 1 and Stage 4 non*-MYCN*-amplified patients is shown in Fig. 1c.

Based on prior work (see [12] for a comprehensive review), we expect candidate MRs to be highly enriched in *MYCN*^Amp^ synthetic lethals. We thus compared the 48 high-confidence MRs with the 25 transcriptional regulators emerging from the pooled shRNA screen (Fig. 1e), which led to identification of four common proteins: transcriptional factor AP4 (*TFAP4*); mitochondrial recycling factor (*MRRF*); protein kinase DNA-activated catalytic polypeptide (*PRKDC*); and zinc finger protein 77 (*ZNF77*). The shRNAs barcodes for these four genes were significantly depleted in the whole-genome shRNA screen of MYCN ON population, with *TFAP4*, *MRRF*, *PRKDC*, and *ZNF77*, ranking 149, 95, 145, 218, respectively (Fig. 1f). Interestingly, these candidate synthetic lethal proteins are identified as significant MRs when comparing Stage 4 *MYCN-*amplified patients with Stage 1 patients (Fig. 1g), but not when comparing Stage 4 (high-risk) non-*MYCN*-amplified patients to the same set of Stage 1 patients (Fig. 1h), suggesting that their activity is *MYCN* amplification-dependent. Among the four candidates, *TFAP4* was the highest ranked candidate in both databases, ranking 11th in the TARGET cohort, and 1st in the NRC cohort (Supplementary Fig. S2), and was thus selected for further experimental and computational validation studies.

### *TFAP4* expression and activity correlates with survival

**Fig. 2:**
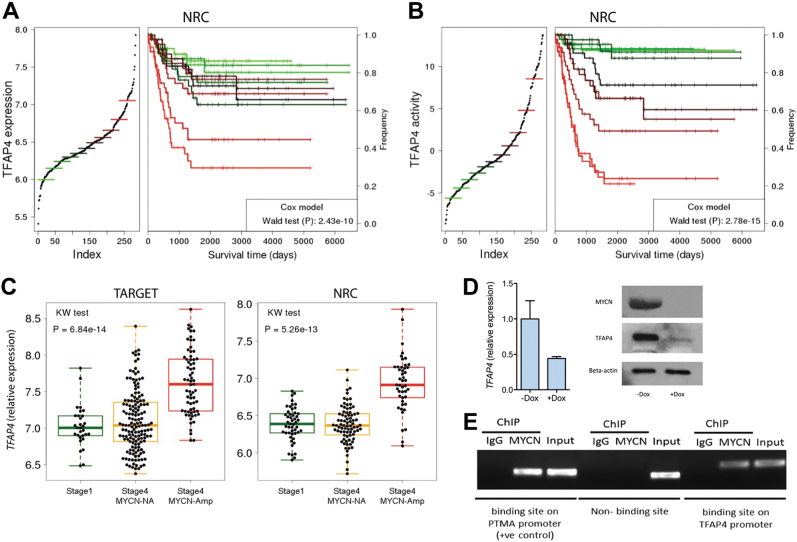
*TFAP4* expression is upregulated by MYCN and is strongly correlated with patient survival. **a** Kaplan–Meier curve depicting corresponding increase in poor outcome with increasing expression of TFAP4. *P* value was calculated using a Cox proportional hazards model after removing stage 1 patient samples. **b** Kaplan–Meier curve depicting corresponding increase in poor outcome with increasing TFAP4 activity. *P* value was calculated using a Cox proportional hazards model after removing stage one patient samples. **c** Box plot of *TFAP4* expression in stage 4 *MYCN-*amplified patients (red), stage 4 non-*MYCN*-amplified patients (yellow) and stage 1 low-risk patients (green). Expression data are from NCI TARGET (left) and NRC (right). **d** *TFAP4* gene expression in SHEP21N cells with or without doxycycline. Gene expression was analyzed by qPCR and immunoblotting 72 h after doxycycline (1 μg/ml) addition. **e** ChIP assay showing that MYCN binds to the predicted binding site in the first intron of *TFAP4*, but not at a non-binding site in *TFAP4*. Chromatin was cross-linked and subjected to ChIP analysis with a MYCN specific antibody and, as a control, rabbit IgG. PCR analysis was performed with primers flanking the first canonical E-box in the first intron of *TFAP4* or a control non-binding primer pair localized in the last intron of *TFAP4*. Primers flanking a canonical E-box in the known MYCN/c-MYC target gene prothymosin α (*PTMA*) [22], was used as a positive control

We performed Cox proportional hazards analysis on the NRC cohort patient samples. We found that *TFAP4* expression (Fig. 2a) and activity (Fig. 2b) are strong negative predictors of patient survival (Wald test, *P* = 2.43E-10, HR = 6.2; Wald test *P* = 2.78E-15, HR = 10.94, respectively). As shown in previous studies [15, 18], activity significantly outperformed expression as a predictor. Multivariate Cox regression analysis showed that *TFAP4* expression represents an independent negative predictor of survival compared with other clinical and biological correlates for risk stratification [24], including stage (*P* = 2.7E-5, HR = 3.28) and age (*P* = 9.8E-6, HR = 3.53), but not *MYCN* amplification (*P* = 1.4E-1, HR = 1.76) (Table 1).

**Table 1:** Cox proportional hazards analysis on the NRC cohort patient samples

	Hazard ratio	CI05	CI95	*P* value
TFAP4	6.22	3.53	10.94	2.43E-10
TFAP4 +stage	3.29	1.89	5.73	2.67E-05
TFAP4 + age	3.54	2.02	6.19	9.79E-06
TFAP4 + MYCN	1.76	0.82	3.78	1.45E-01
TFAP4 + stage + age + MYCN	1.72	0.79	3.74	1.68E-01

CI05 = Confidence Interval 0.05

CI95 = Confidence Interval 0.95

Examining the TARGET and NRC data sets, we found that *TFAP4* expression is significantly higher in Stage 4 *MYCN-*amplified patients (Fig. 2c) compared with both Stage 4 non-*MYCN*-amplified patients (*P* = 1.05E-13, TARGET cohort; *P* = 8.44E-13, NRC cohort) and Stage 1 low-risk patients (*P* = 2.54E-12, TARGET cohort; *P* = 6.01E-12, NRC cohort).

### TFAP4 is regulated by MYCN

TFAP4 is a ubiquitously expressed transcription factor belonging to the basic helix-loop-helix leucine zipper group of proteins and has previously been shown to be a direct and conserved target of MYC [25]. Assessing the regulation of TFAP4 by MYCN/MYC, we found that high *TFAP4* expression correlates with high *MYCN* or *MYC* expression in neuroblastoma cell lines and in the TARGET cohort of patients (Supplementary Fig. S3A, B, C). To demonstrate that TFAP4 is regulated by MYCN, we examined the SHEP21N cell line. When *MYCN* expression is switched off by doxycycline, *TFAP4* expression is markedly decreased (Fig. 2d). Knockdown of MYCN in the *MYCN*-amplified neuroblastoma cell line SK-N-BE(2) also resulted in decreased expression of *TFAP* (Supplementary Fig. S4), supporting the notion that MYCN regulates *TFAP4* expression.

Jung et al. [25] have previously identified four canonical MYC E-box-binding sites (CACGTG) in intron 1 of human *TFAP4*. We performed a ChIP-PCR assay with primers flanking the first putative MYC/MYCN E-box binding (+660 relative to the transcriptional start site), and an anti-MYCN antibody. ChIP-PCR assay demonstrated that MYCN directly binds to the predicted binding site (Fig. 2e), indicating that TFAP4 is directly regulated by MYCN. Consistent with our results, Hsu et al. [26], performed ChIP-seq using an anti-MYCN antibody in the MYCN-amplified neuroblastoma cell BE(2)C, demonstrated that TFAP4 is a direct transcriptional MYCN target.

### Silencing *TFAP4* selectively inhibits growth of *MYCN-*amplified neuroblastoma cell lines

**Fig. 3:**
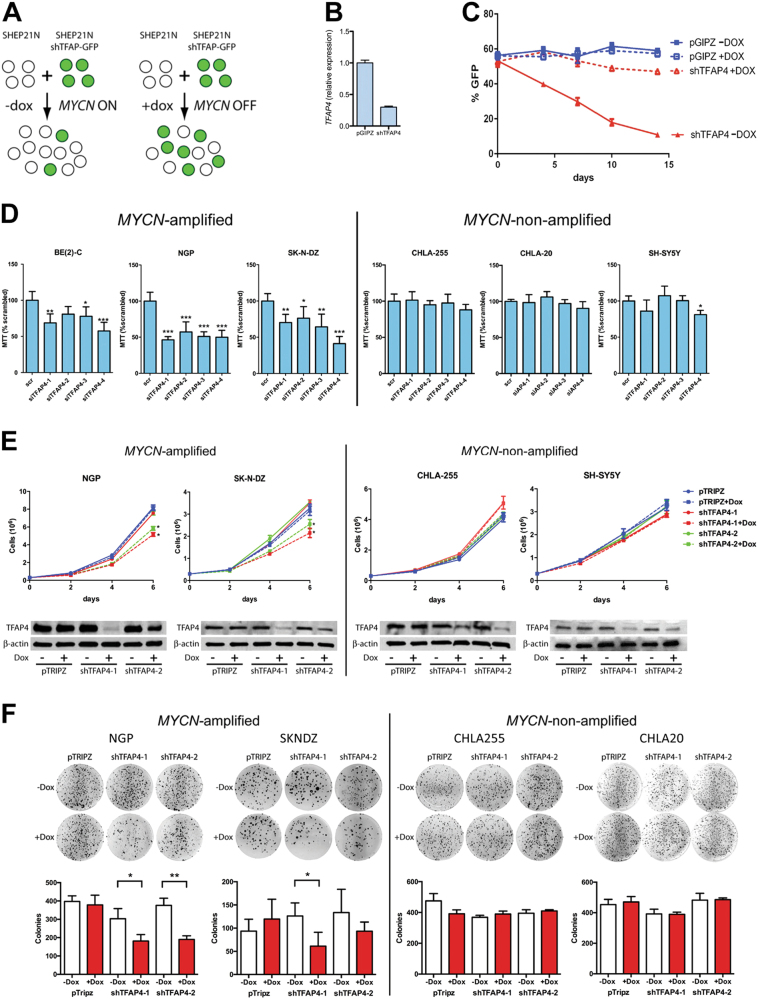
Silencing *TFAP4* selectively inhibits growth of *MYCN-*amplified neuroblastoma cell lines. **a** Schema of cell growth competition assay. SHEP21N cells infected with either shTFAP4-GFP or an empty vector control pGIPZ-GFP were mixed with equal number of uninfected SHEP21N cells. The infected cells to WT cells ratio is measured by FACS. **b** *TFAP4* gene expression (qRT-PCR) in SHEP21N-pGIPZ control cells and SHEP21N expressing shTFAP4. **c** %GFP ratio measured at day 4, 7, 10, and 14. Experiments were performed in triplicate. Mean ± std dev. **d** % of neuroblastoma cell survival 96 h after siRNA-mediated downregulation of *TFAP4*. Neuroblastoma cell lines were transfected with four different constructs of siRNA against *TFAP4*. The experiments were performed in quadruplicate. Mean ± std dev. *, *P* < 0.05; **, *P* < 0.01; ***, *P* < 0.001. **e** Proliferation assays showing neuroblastoma cell growth after silencing TFAP4 by doxycycline-inducible shRNAs. Neuroblastoma cell lines were infected with two different dox-inducible shRNAs against *TFAP4* as well as the empty vector control pTRIPZ. shRNA was induced by 1 μg/ml doxycycline at day 0. Cells were counted on day 0, 2, 4, and 6. Experiments were performed in triplicate. Mean ± std dev. *, *P* < 0.05; *TFAP4* silencing was confirmed by western blot. **f** Colony formation assay. Neuroblastoma cells infected with doxycycline-inducible shRNAs were plated in semisolid agar media for 21 days and stained with MTT. Experiments were done in triplicate. Mean ± std dev. *, *P* < 0.05; **, *P* < 0.01

To validate TFAP4 synthetic lethality with *MYCN* amplification, we performed a multicolor competition assay with SHEP-21 N cell line (Fig. 3a). SHEP-21 N was infected with a TFAP4 shRNA-GFP vector or the empty vector pGIPZ-GFP. Silencing of TFAP4 was confirmed (Fig. 3b). An equal number of GFP(+) SHEP21N cells and uninfected GFP(−) SHEP-21 N cells were mixed together, and the percentage of GFP(+) cells determined by fluorescence-activated cell sorting (FACS) with (MYCN OFF) or without doxycycline (MYCN ON) (Fig. 3c). When MYCN was ON (-DOX), silencing of TFAP4 markedly decreased the percentage of GFP(+) cells, which demonstrate that silencing TFAP4 inhibits cell proliferation only when *MYCN* expression is high.

To further validate synthetic lethality of TFAP4 to *MYCN* amplification, we examined three *MYCN-*amplified human neuroblastoma cell lines (BE(2)C, NGP and SK-N-DZ) and three non-*MYCN*-amplified human neuroblastoma cell lines (CHLA-255, CHLA-20, and SH-SY5Y). Four different constructs of siRNAs were used to silence TFAP4 and compared with scrambled siRNA (Supplementary Fig. S5). After 96 h of siRNA transfection, silencing *TFAP4* significantly reduced survival of *MYCN-*amplified cell lines by an average of 49% in NGP, 37% in SK-N-DZ, and 32% in BE(2)C, whereas cell survival of non-*MYCN*-amplified lines CHLA-255, CHLA-20, and SH-SY5Y, was unaffected (Fig. 3d).

To evaluate the long-term effects of silencing *TFAP4*, we generated stable cell lines with doxycycline-inducible shRNAs against *TFAP4*. Silencing *TFAP4* significantly reduced growth of *MYCN-*amplified NGP and SK-N-DZ cells by 25% and 35%, respectively, at 6 days after shRNA induction, whereas growth of non-*MYCN*-amplified CHLA-255 and SH-SY5Y cells was not affected (Fig. 3e). We further validated the results by the soft agar colony formation assay. Silencing of *TFAP4* significantly reduced colony formation in NGP by 47% and SK-N-DZ by 43% after 3 weeks, whereas there was no difference in CHLA-255 and CHLA-20 colony formation (Fig. 3f, Supplementary Fig. S6).

Cell cycle profiling of neuroblastoma cells lines showed that silencing TFAP4 increased the percentage of cell population in G0/G1 phase in NGP and SK-N-DZ by 9%, but had no effect on CHLA-255 and SH-SY5Y (Supplementary Fig. S7). Silencing TFAP4, however, did not induce apoptosis in either *MYCN-*amplified or non-*MYCN*-amplified cell lines (Supplementary Fig. S8). Overall, these results demonstrate that TFAP4 regulates proliferation in *MYCN-*amplified neuroblastoma.

### Silencing *TFAP4* induces neuroblastoma differentiation

**Fig. 4:**
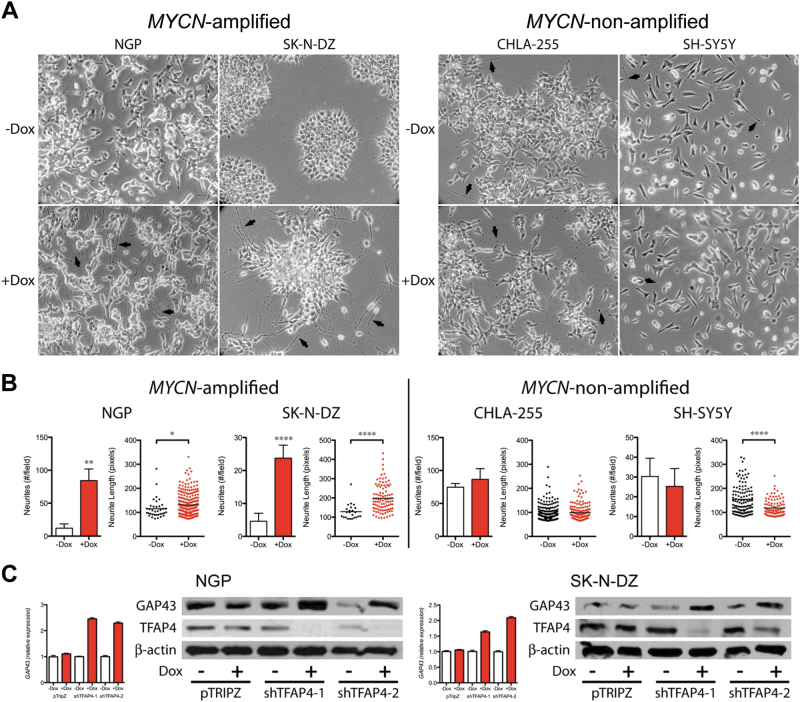
Silencing *TFAP4* induces neuroblastoma cell differentiation. **a** Morphology of neuroblastoma cells after silencing *TFAP4*. Cells were grown in culture for 8 days, with 1 μg/ml doxycycline (shRNA induced) or without doxycycline. **b** Neurites # per field, and neurite length (pixels) were quantified with or without doxycycline. Mean ± std dev. *, *P* < 0.05; **, *P* < 0.01, ****, *P* < 0.0001. **c** *GAP43* expression level (qRT-PCR) and protein level (western blot) in neuroblastoma cells. Gene expression was measured by quantitative PCR 4 days after induction of shRNA against *TFAP4*. Protein samples for western blot were collected from *MYCN-*amplified cells that were cultured with or without doxycycline (1 μg/ml) for 6 days

After 8 days of doxycycline treatment, we observed morphologically increased neurite outgrowth from both NGP and SK-N-DZ cells, suggesting that the cells were undergoing neuronal differentiation (Fig. 4a). Quantification demonstrated that silencing of TFAP4 significantly increased neurites/field and neurite length in *MYCN-*amplified cell lines (Fig. 4b; sevenfold and 5.2-fold increase in mean neurites/field, NGP and SK-N-DZ, respectively; 14% and 55% increase in mean neurite length, NGP and SK-N-DZ, respectively), but not in non-*MYCN*-amplified cell lines (Fig. 4b). Growth-associated protein 43 (GAP43) is a neuron-specific protein found in growth cones, whose expression is regulated during neuronal differentiation [27]. Silencing of TFAP4 increased expression of *GAP43*, 2.4-fold in NGP and 1.9-fold in SK-N-DZ as determined by qPCR (Fig. 4c), which was confirmed by western blot (Fig. 4c). The neuronal marker tyrosine hydroxylase (TH) was also increased 1.9-fold in NGP and 1.6-fold in SK-N-DZ upon silencing of TFAP4 (Supplementary Fig. S9). Supporting the inverse correlation of TFAP4 and neuronal differentiation, analysis of the TARGET data set demonstrated that tumors with the highest *TFAP4* expression had significantly lower expression of *GAP43* (Supplementary Fig. S10).

### Silencing *TFAP4* selectively inhibits growth of *MYCN-*amplified neuroblastoma xenograft tumors

**Fig. 5:**
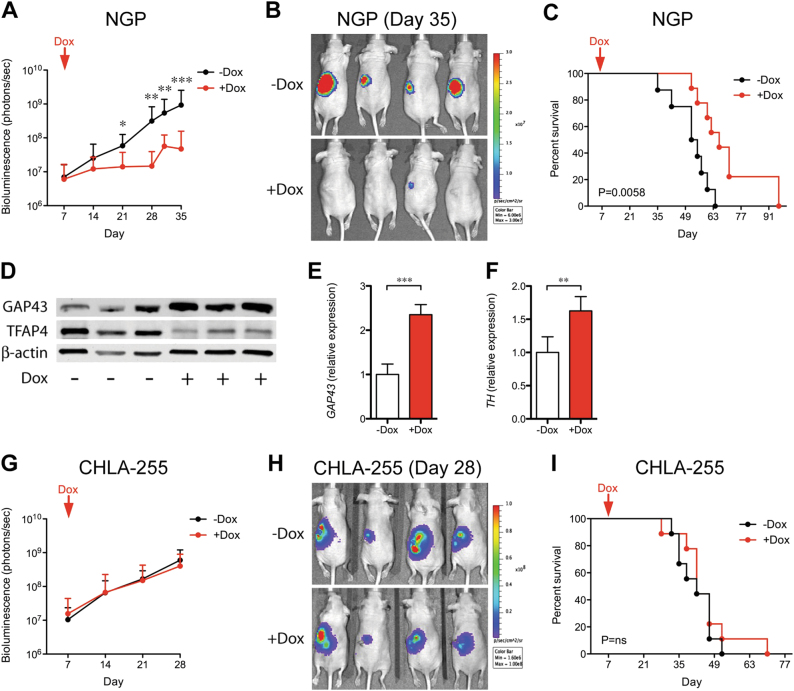
Silencing *TFAP4* selectively inhibits growth of *MYCN-*amplified neuroblastoma xenograft tumors. **a** Luciferase activity of the NGP tumor over time. *, *P* < 0.05; **, *P* < 0.01. 10^6^ luciferase-labeled neuroblastoma cells with dox-inducible shTFAP4 were implanted into the nude mice. Mice were randomized and half were given drinking water with doxycycline (+ Dox, *n* = 10) and without (− Dox, *n* = 9). **b** Representative bioluminescence imaging at Day 35. **c** Kaplan–Meier curve of mice with TFAP4-silenced (+ Dox) or control (− Dox). Mice were killed when luciferase activity reached 6 × 10^9^ photons/sec. *P* = 0.0058. **d** Protein level of the neuronal marker GAP43 in NGP tumors. **e** *GAP43* expression in control (− Dox) or TFAP4-silenced (+ Dox), tumors. Mean ± std dev. ***, *P* < 0.001. **f** *TH* expression in control (− Dox) or TFAP4-silenced ( + Dox), tumors. Mean ± std dev. **, *P* < 0.001. **g** Luciferase activity of CHLA-255 tumors over time, (− Dox, *n* = 9; + Dox, *n* = 9). **h** Representative bioluminescence imaging at Day 28. **i** Kaplan–Meier curve of mice with TFAP4-silenced CHLA-255 ( + Dox) or control (− Dox). *P* *=* n.s

We next validated TFAP4 synthetic lethality with *MYCN* amplification in a xenograft model. In total, 10^6^ luciferase-expressing NGP shTFAP4 cells were implanted intrarenally in nude mice. Mice were randomized after 7 days, and half the mice were given drinking water with doxycycline to induce silencing of *TFAP4*. From day 21 onwards, we observed significantly reduced tumor growth in the *TFAP4*-silenced group (+ Dox) compared with the control (− Dox) (Fig. 5a, b). Survival was defined as the day that the primary tumor luciferase flux reached 6 × 10^9^ photons/sec. Silencing of *TFAP4* significantly increased median survival by 12.5 days (Fig. 5c; 66.0 vs. 53.5 days, + Dox vs. − Dox, respectively, *P* = 0.0058). There was no difference in tumor weight between the two groups, suggesting that the primary tumor luciferase flux was correlated with the tumor weight (Supplementary Fig. S11). There was also no difference in bioluminescence reading in circulating tumor cells, bone marrow, or liver between the two groups (Supplementary Fig. S12A–C), indicating that silencing *TFAP4* did not affect metastasis. Decreased TFAP4 expression in + Dox tumors was verified by western blot (Fig. 5d), indicating that tumor progression was not due to escape from silencing of TFAP4. Tumors in which TFAP4 was silenced demonstrated an increase in markers of neuronal differentiation, with increased GAP43 by western blot (Fig. 5d), 2.4-fold by qPCR (Fig. 5e), and increased TH 1.6-fold by qPCR (Fig. 5f). We also validated the effect of silencing *TFAP4* in *MYCN-*amplified SK-N-DZ tumors, using the same intrarenal xenograft model. We also observed significantly reduced tumor growth in the *TFAP4*-silenced group (+ Dox) compared with the control (− Dox), from day 31 onwards (Supplementary Fig. S13).

To determine whether silencing TFAP4 could inhibit the growth of established tumors, NGP tumors were allowed to grow for 24 days, before treating with doxycycline to silence TFAP4. Mice were killed when the primary tumor flux reached 6 × 10^9^ photons/sec. Although there was no difference in tumor flux for the initial days following addition of doxycycline (Supplementary Fig. S14A), silencing of TFAP4 in NGP tumors significantly prolonged overall survival by 13 days (Supplementary Fig. S14B; median survival 86 vs. 73 days, + Dox vs. − Dox, respectively *P* = 0.03).

We also determined whether silencing of *TFAP4* would affect the growth of a non-*MYCN*-amplified xenograft. CHLA-255 shTFAP4 cells were implanted intrarenally, and doxycycline treatment started 7 days later (Fig. 5g, h). Silencing of TFAP4 had no effect on tumor growth as assessed by bioluminescence (Fig. 5g) or on survival (Fig. 5i). *TFAP4* silencing in tumors was confirmed by western blot (Supplementary Fig. S15). We repeated the experiment with established CHLA-255 tumors and found that silencing of TFAP4 did not affect CHLA-255 growth or overall survival (Supplementary Fig. S14 C, D).

### Identification of genes regulated by TFAP4 in *MYCN*-amplified neuroblastoma

To identify genes that are regulated by TFAP4 in *MYCN-*amplified neuroblastoma, we profiled genome-wide transcriptional changes by RNAseq 40 h after shRNA against *TFAP4* was induced. Gene set enrichment analysis (GSEA) analysis demonstrated that the expected TFAP4 targets were downregulated upon TFAP4 knockdown (Gene set: CAGCTG_V$AP4_Q5, FDR 6.70E-08), validating the experiment. The rest of the analysis, however, identified only a few pathways (Supplemental Fig. S16), that did not appear related to TFAP4 function. We, therefore, opted to examine the expression of individual genes that were altered with silencing of TFAP4.

**Fig. 6:**
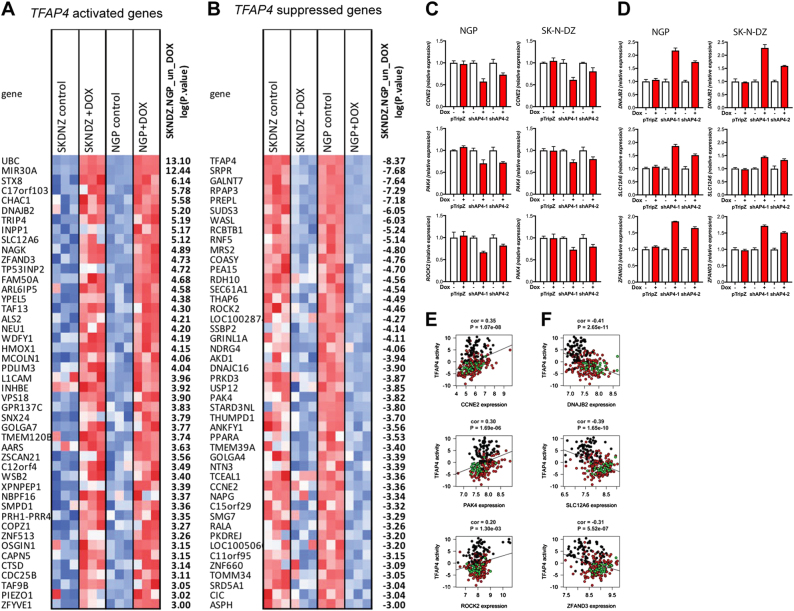
Genes regulated by TFAP4 in *MYCN-*amplified neuroblastoma. Differentially expressed genes in *MYCN-*amplified neuroblastoma after silencing TFAP4. Forty hrs shRNA induction by doxycycline, total mRNA was collected from NGP and SK-N-DZ and analyzed by RNAseq. mRNA most significantly altered by shTFAP4 induction were shown (*P* < 0.001). **a** genes activated by TFAP4, **b** genes repressed by TFAP4. Relative expression of TFAP4-activated genes **c** and TFAP4 repressed genes **d** measured by qRT-PCR. Gene expression was measured by quantitative PCR 48 h after induction of shRNA against *TFAP4*. Mean ± std dev. Scatter plot and correlation analysis between TFAP4 VIPER activity and *TFAP4*-activated genes **e** and repressed genes **f**

Silencing TFAP4 downregulated the expression of 457 genes (e.g., TFAP4-activated genes), and upregulated 415 genes (e.g., TFAP4-suppressed genes). The most significantly differentially expressed genes (*P* *<* 0.001), are shown in Fig. 6a, b. We validated TFAP4-activated genes by qPCR: Cyclin E2 (*CCNE2*; ranked 34th of 14893 genes analyzed in RNA sequencing profiling) involved in G1/S cell cycle progression; *PAK4* (ranked 25th) and Rho-associated coiled-coil containing kinase 2 (*ROCK2;* ranked 16th) involved in focal adhesion and axon guidance. Silencing *TFAP4* reduced *CCNE2, ROCK2*, and *PAK4* expression in both NGP and SK-N-DZ (Fig. 6c). We also observed a significant positive correlation between the TFAP4 single sample VIPER activity and *CCNE2, ROCK2*, and *PAK4* expression (Fig. 6e).

We also validated TFAP4-suppressed genes by qPCR: DnaJ Heat Shock Protein Family (Hsp40) Member B2 (*DNAJB2*, ranked 5th) a co-chaperone regulator of Hsp70 that is expressed principally in the nervous system, Solute Carrier Family 12 Member 6 (*SLC12A6*, ranked 8th) a member of the K-Cl cotransporter (KCC) family, and Zinc Finger AN1-Type Containing 3 (*ZFAND3*, ranked 7th), which is expressed preferentially in post-meiotic cells in the testis during spermatogenesis. Silencing *TFAP4* increased *DNAJB2, SLC12A6*, and *ZFAND3* expression in both NGP and SK-N-DZ (Fig. 6d). We also observed a significant negative correlation between the TFAP4 single sample VIPER activity and *DNAJB2, SLC12A6*, and *ZFAND3* expression (Fig. 6f).

## Discussion

Although *MYCN* amplification has been identified as a marker of poor prognosis for neuroblastoma, no therapeutic drug has been developed to target MYCN directly. Here we combined whole-genome shRNA library screening and computational master regulator inference analysis to identify novel drug targets for *MYCN-*amplified neuroblastoma. Compared with previous MYC/MYCN driven synthetic lethal screens, our approach has several advantages. (1) We used a whole-genome shRNA library rather than a subset of shRNAs. This unbiased selection of library allowed us to discover novel synthetic lethal interactors whose function was previous not well studied. (2) MARINa analysis is an algorithm that interrogates patient expression databases and predicts a short list of master regulators that drives development of a certain disease phenotype. Correlating the functional shRNA screen results with analysis of master regulators of *MYCN* amplification neuroblastoma allowed us to overcome the limitations of in vitro shRNA screening and provided a robust short list of synthetic lethal master regulators. We identified four MRs: TFAP4, MRRF, PRKDC, and ZNF77. Among them, PRKDC was previously validated as a synthetic lethal gene discovered in a screen with kinase shRNA pool in MYC-overexpressed cells [8], a finding that supports the robustness of our approach.

We selected TFAP4 as our top candidate. Reports have shown that TFAP4 regulates a number of biological processes including cell cycle [25, 28], senescence [29], immune response [30], epithelial to mesenchymal transition [31], and maintenance of stemness [29, 32]. Here, we demonstrate that TFAP4 is a direct target of MYCN, and that its expression is significantly upregulated in stage 4 *MYCN-*amplified neuroblastoma. We further validated synthetic lethality of TFAP4 with *MYCN* amplification in vitro and in vivo in a range of neuroblastoma cell lines. Our results are consistent with those of Xue et al. [33], who reported that silencing of TFAP4 inhibited proliferation of *MYCN*-expressing cell lines in vitro.

Mechanistically, we observed that in *MYCN-*amplified neuroblastoma cells, silencing of *TFAP4* induces neurite outgrowth and upregulation of the neuronal marker GAP43. Consistent with this observation, TFAP4 has been reported to inhibit differentiation, as TFAP4 upregulated the expression of two colorectal cancer stem cell markers, CD44 and LGR5 [29]. TFAP4 levels were found to gradually decline during development, from embryonic to adult brain [32]. In nonneuronal cells, TFAP4 forms a repressor complex with Geminin and HDAC3 to downregulate the neuronal gene *PAHX-AP1* [32]. TFAP4 has also been shown to represses the neuronal DBH genes by interaction with GATA-3 and Sp1 [34].

Cells undergoing differentiation frequently display slower cell cycle progression. TFAP4 has been shown to repress the cell cycle checkpoint gene *CDKN1A* in a breast cancer cell line MCF7 [25]. Our cell cycle profiling results show that silencing TFAP4 inhibited G1 cell cycle progression. We validated that one of the top TFAP4 targets *CCNE2*, encoding cyclin E2 protein, is activated by TFAP4. Cyclin E2 partners with CDK2 at the G1/S checkpoint, and overexpression accelerates G1 cell cycle progression [35]. Thus, TFAP4 may contribute to accelerating cell cycle progression via upregulation of *CCNE2*.

Genes involved in blocking differentiation frequently function in epithelial to mesenchymal transition (EMT). Pathways involved in EMT promote the loss of cell adhesion and gain migratory abilities [36]. A genome-wide expression study showed that numerous genes implicated in EMT were differentially regulated by TFAP4 [37]. Among them, the regulation of the EMT-associated genes *CDH1* and *SNAIL* were further validated in colorectal cancer cell lines DLD-1 and HT29. We showed that TFAP4 upregulates the top two genes involved in epithelial to mesenchymal transition, *PAK4* and *ROCK2*. Both ROCK2 and PAK4 are signaling molecules that function in cytoskeleton rearrangement and cell migration [38, 39]. Their expression is frequently upregulated in metastatic cancers [40–42]. Moreover, silencing of *PAK4* has been shown to reduce neuronal progenitor cell proliferation and self-renewal ability [43]. ROCK2 has also been shown to inhibit neuronal differentiation in neuroblastoma, as both knockdown of *ROCK2* [44], and a novel ROCK2 inhibitor [45] increases neurite outgrowth. Although *SNAIL* and *CDH1* were not identified in our RNAseq analysis, it is possible that TFAP4 regulates a different set of EMT effectors and may restrict differentiation of *MYCN*-amplified neuroblastoma by this mechanism as well.

Taken together, we propose a model that posits that, in *MYCN-*amplified neuroblastoma, high levels of MYCN upregulate the master regulator TFAP4, which in turn activates downstream oncogenic signaling pathways to promote cell cycle progression and inhibit neuroblastoma differentiation. TFAP4 and MYCN may regulate different sets of genes involved in these processes. As a result, TFAP4 may play additional oncogenic roles in *MYCN-*amplified neuroblastoma tumorigenesis. As *TFAP4-*null mice are normal and fertile, TFAP4 is not essential for development or normal physiological function. Thus, TFAP4 is an attractive therapeutic target, as it is both synthetically lethal and a master regulator for *MYCN-*amplified neuroblastoma.

## Materials and methods

### Cell culture

Neuroblastoma cell lines NGP and SHEP-21 N (gift of Dr. Garrett Brodeur), SH-SY5Y (ATCC) were cultured in Rosewell Park Memorial Institue medium (Gibco) supplemented with 10% fetal bovine serum (FBS) and 1% penicillin/streptomycin (Invitrogen). Neuroblastoma cell lines SK-N-SH, BE(2)C, SK-N-DZ (ATCC), CHLA-20, and CHLA-255 (gift of Dr. C. Patrick Reynolds) were cultured in Dulbecco’s Modified Eagle Medium (Gibco) with 10% FBS and 1% penicillin/streptomycin. Cell lines were incubated humidified at 37˚C and 5% CO_2_. All cell lines were authenticated by short tandem repeat profiling. Cells were checked routinely for the absence of mycoplasma.

### Whole-genome shRNA screen

SHEP-21 N cells were infected with a whole-genome pGIPZ lentiviral shRNA-mir library (gift of Dr. Jose Silva) in independent duplicates. Antibiotic selection was initiated 3 days after infection with 1μg/ml puromycin (Sigma). After selection, each replicate was divided into two populations and cultured in the absence or presence of 1 µg/ml doxycycline for 10 doubling times. Genomic DNA was extracted from the cell populations, and the shRNA regions were PCR amplified. The PCR products were hybridized to a customized microarray (Agilent). Statistical analysis method of the microarray data is described in ref. [23].

### Master regulator inference analysis

The two databases used in this study are the NCI TARGET database (http://target.nci.nih.gov), and the European Neuroblastoma Research Network database (https://www.siopen-r-net.org/). Patient-derived gene expression profiles were analyzed using ARACNe (Algorithm for the Reconstruction of Accurate Cellular Networks) algorithm to infer a neuroblastoma-specific transcriptional interaction network [46]. Differential expression signatures were then calculated by comparing *MYCN*-amplified samples against Stage 1 patients. The VIPER algorithm was used to compute the normalized enrichment score (NES) and the master regulators were then ranked according to their NES [15].

### Cell competition assay

SHEP21N cells were infected with pGIPZ-shTFAP4 (Dharmacon V3LHS_301156) or pGIPZ control plasmid. The GFP + pGIPZ-shTFAP4 or the pGIPZ control SHEP21N cells were mixed in equal amounts with the parental SHEP21N cells (GFP − ) and plated in 10 cm tissue culture plates. Cells were collected at various time points and percentage of GFP in the population was analyzed by FACS analyzer (LSRII Flow cytometer, BD).

### Cell proliferation assays

Neuroblastoma cells were transiently transfected with 50 nM siRNA (Dharmacon LQ-009504-00-0002) packed in DharmaFECT reagents (Dharmacon #T-2001-01). The transfected cells were then seeded into 96-well plates. Cells were stained with 10 µg/ml fluorescein diacetate and 0.1% Eosin Y (Sigma) 96 h after transfection. Fluorescence from stained live cells was measured by Dimscan, as described in ref. [47].

### Colony formation assays

Neuroblastoma cells with shRNAs (Dharmacon V3THS_301154, V3THS_301158) were plated in semisolid media as follows in six-well plates: 0.6% agar (Fisher Scientific), neuroblastoma cells infected with pTRIPZ-shRNA in 0.3% agar, and appropriate media with or without doxycycline (1 μg/ml). Cells were re-fed with 2 ml medium every 2 days until colonies were macroscopic. The colonies were stained with 1 mg/ml Thiazolyl Blue Tetrazolium Bromide (Sigma). Photos of the stained colonies were taken and pixels of colonies were quantified using Adobe Photoshop software.

### TUNEL assay

Neuroblastoma cells with pTRIPZ-shRNA were plated on 96-wells plate. Apoptosis was evaluated by TUNEL three days after doxycycline induction, using the HT TiterTACS colorimetric apoptosis detection kit (Trevigen #4822-96-K).

### Western blot

Cells were lysed in protein lysis buffer (× 10 RIPA buffer (Sigma), 1 mM phenylmethylsulfonyl fluoride and protease inhibitor cocktail (Sigma). Tumor tissue was snap-frozen in liquid nitrogen then homogenized in the same protein lysis buffer. The following antibodies were used for western blotting: anti-MYCN (1:200; Santa-Cruz Biotechnology #sc-53993), anti-TFAP4 (1:200; Santa-Cruz Biotechnology #sc-18593), anti-MYC (1:200, Santa-Cruz Biotechnology #sc-40), anti-GAP43 (1:20000; Novus Biologicals #NB300-143), and anti-β-actin (1:5000; Cell Signaling #4967 S).

### RNA extraction and quantitative real-time PCR

Total RNA was isolated from cell lines or snap-frozen xenograft tissue using the RNEASY mini kit (Qiagen #74106). The Verso cDNA Kit (Thermo Scientific #AB1453A) was used to transcribe cDNAs. Relative gene expression was measured on ABI 7300 real-time PCR System. Sequence of the primers or catalog number of the commercial primers are listed in Table 2.

**Table 2:** Primer sequences for real-time PCR

Oligonucleotides	
*ACTB*-For	ATTGGCAATGAGCGGTTC
*ACTB*-Rev	CGTGGATGCCACAGGACT
*CCNE2*-For	GGGAAACATTTTATCTTGCACA
*CCNE2*-Rev	CTGCAAGCACCATCAGTGAC
*PAK4*-For	CCACCGGGACATCAAGAG
*PAK4*-Rev	CAGAACCCAAAGTCTGACAGC
*ROCK2*-For	CGCTGATCCGAGACCCT
*ROCK2*-Rev	TTGTTTTTCCTCAAAGCAGGA
*MYCN*-For	GCACAGACTGTAGCCATCCG
*MYCN*-Rev	TTTAATACCGGGGGTGCTTCC
TFAP4-For	AGGTCTCCGTTGCTTCTTG
TFAP4-Rev	GGAGAGTGGCGAATTCTAGTG
Pre-designed TaqMan® Assay Primer/Probe Sets	
*CYC*	Applied Biosystems 4326316E
*DNAJB2*	Applied Biosystems Hs01047948_m1
*GAP43*	Applied Biosystems Hs00967138_m1
*HPRT1*	Applied Biosystems 4326321E
*MYC*	Applied Biosystems Hs00153408_m1
*MYCN*	Applied Biosystems Hs00232074_m1
*SLC12A6*	Applied Biosystems Hs00994548_m1
*TFAP4*	Applied Biosystems Hs01558245_m1
*TH*	Applied Biosystems Hs00165941_m1
*ZFAND3*	Applied Biosystems Hs00938278_m1

### Neurite quantification

Neuroblastoma cells lines with pTRIPZ-shRNA were grown in culture for 8 days. Images at × 20 were obtained and individual neurites were visually identified and traced using Adobe Photoshop software. Neurite length was quantified, with those less than a cell body diameter excluded.

### Chromatin immunoprecipitation

NGP cells were processed following manufacturer’s instruction using truChIP chromatin shearing kit (Covaris #520154). Chromatin was sheared by sonication (Covaris, S-220 series). Pre-clearing, incubating with antibodies, and reversal of cross-linking was performed with EZ-ChIP immunoprecipitation kit (Millipore #17-371). Anti-MYCN antibody (Santa-Cruz Biotechnology #sc-53993) and rabbit anti-mouse IgG (Sigma #M7023) were used.

Primer sequences (5′–3′) used for PCR amplification (*TFAP4* primers from Jung et al., [25]) are listed in Table 3.

**Table 3:** Primer sequences for chromatin immunoprecipitation

Name	Position relative to the transcriptional start site (+1)	Sequence (5′–3′)
*TFAP4* intron 1 Fwd	+582	CGCGACGTTTGTAAATTGC
*TFAP4* intron 1 Rev	+726	CTCAGATCCCGAGGAAGGA
*TFAP4* intron 6 negative control Fwd	+14760	TCTCAGTGGTTCGTCCCTGT
*TFAP4* intron 6 negative control Rev	+14861	GGAGGCGGTGTCAGAGGT
*PTMA*-positive control Fwd	-	ATCTTGTGTGGCACAGGT
*PTMA*-positive control	-	TCGTCTCTGGAGCCAGTTGG

### Animal experiments

NGP and CHLA-255 cells were infected with pTRIPZ-shRNA and fuw-luc plasmid, which constitutively expresses luciferase. Athymic mice, 4–6 weeks of age (Taconic) were anesthetized with ketamine and xylazine, an incision made at the left flank, and 10^6^ cells injected into the renal parenchyma [48]. Seven days or 24 days after implantation, mice were randomized and half of the mice were giving drinking water with 2 mg/ml doxycycline. No blinding was done. Luminescence of tumor cells was measured twice a week using a bioluminescence imaging system (Xenogen) until luminescence reaches threshold of 6 × 10^9^ photons/sec. All procedures were approved by the Institutional Animal Care and Use Committee (IACUC) of Columbia University (Protocol number AC-AAAM5603). Sample sizes were chosen based on our previous experience and in compliance with the protocol.

### Immunohistochemistry staining

Tissue serial section of 5 µm were deparaffinized, rehydrated, and heat-mediated antigen retrieval was performed using DAKO solution. Tissue was stained with anti-TFAP4 primary antibody (1:100; Thermo Scientific #PA5-40702) and appropriate secondary antibodies (Vector).

### RNA sequencing

Cells were treated with or without doxycycline (1 µg/ml) for 40 h. Quality of mRNA was analyzed by bioanalyzer (Agilent). RNA sequencing was conducted at the Columbia Genome Center (http://genomecenter.columbia.edu) using the Illumina platform, and performed at 30 million reads per sample. RNAseq libraries were aligned to hg19 human genome using TopHat. Raw counts of each gene were generated using GenomicFeatures R-system package (Bioconductor). Data were transformed/adapted for linear modeling using voom and differential expression calculated using the limma Bioconductor R package as previously described [49]. The data are available at Gene Expression Omnibus with accession code GSE84389.

We used GSEA in order to calculate differentially active pathways and gene sets. Gene set collections where obtained from MSIG database [50].

### Statistical analysis

Each experiment was performed at least three times. Values are expressed as mean ± SD. Means for the in vitro experiments were compared using two-tailed *t* test or one-way analysis of variance. For each experiments, the exact sample size, the number of replicates, and the statistical results were stated in the result section. Sample sizes and statistical analysis were chosen based on our previous experience and recommendation of biostatistician. Statistical analysis for the in vitro and in vivo experiments were performed using Prism5 software (GraphPad). Survival of neuroblastoma xenograft models was determined by Log-rank (Mantel–Cox). Survival analysis in the NRC cohort was performed using the survival R package.

### Code availability

The VIPER and msVIPER algorithms is available as an R package in Bioconductor (https://www.bioconductor.org/packages/release/bioc/html/viper.html)

## Electronic supplementary material

Supplementary Figures

Supplementary table S1
