# Supplementary material for: Transcription factor activating protein 4 is synthetically lethal and a master regulator of MYCN-amplified neuroblastoma
Source: Oncogene. 2018 Jun 7;37(40):5451–65. doi: 10.1038/s41388-018-0326-9 (PMC6172192; doi:10.1038/s41388-018-0326-9)
Supplement: Supplementary file 1 — Supplementary Figures [file 41388_2018_326_MOESM1_ESM.docx]

**Supplementary Figure S1**

| **A**  **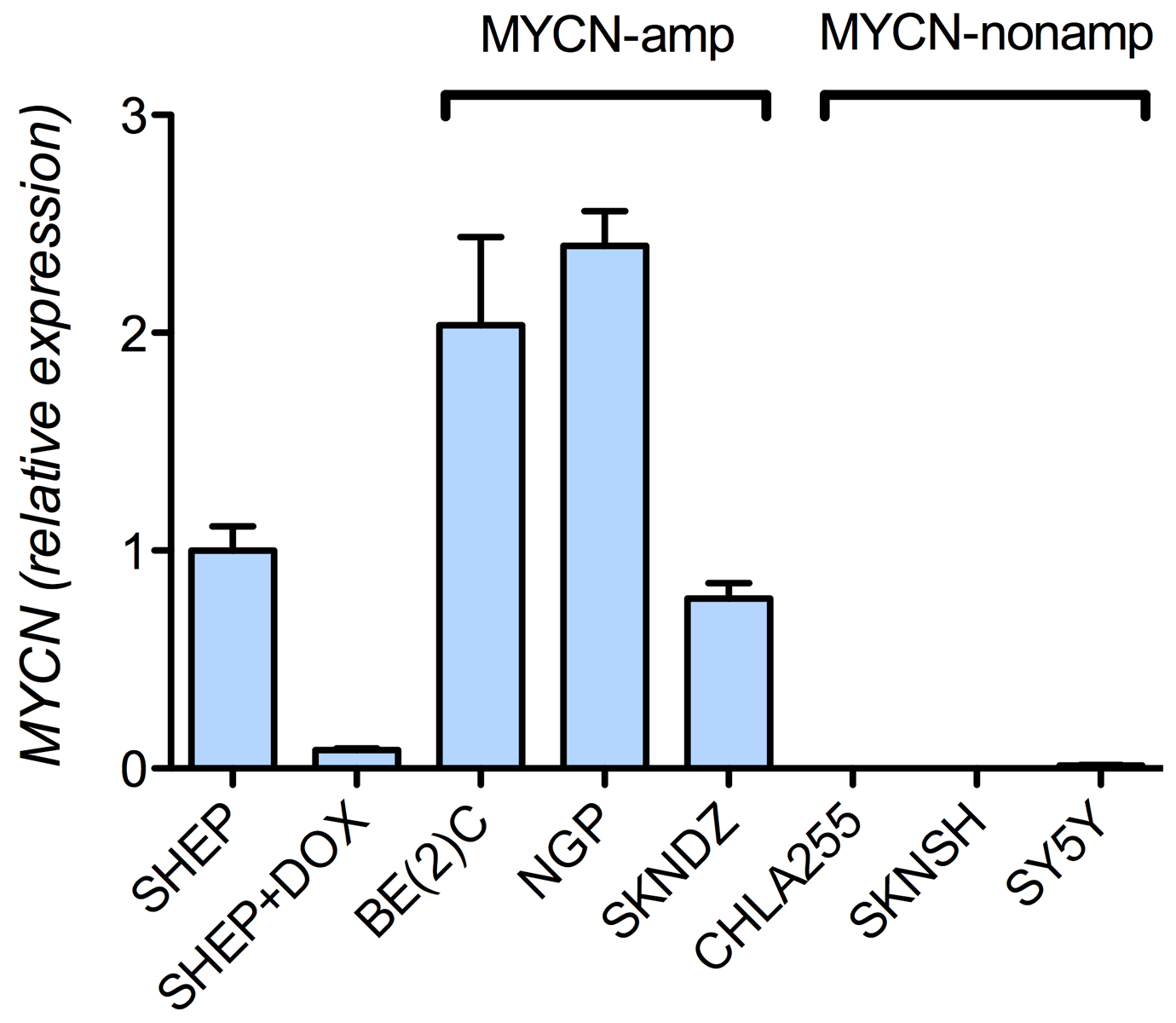** | **B**  **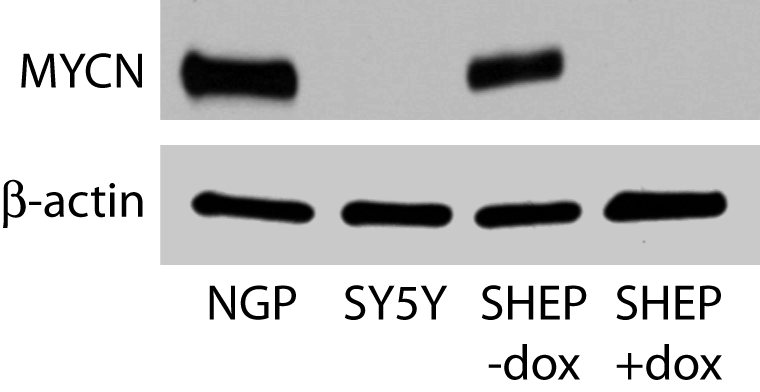** |
| --- | --- |

**Fig. S1. Doxycycline regulates expression of *MYCN* in SHEP‑21N.** **(A)** QPCR showing *MYCN* is highly expressed in SHEP‑21N and in the *MYCN* amplified cell lines BE(2)C, NGP, and SK-N-DZ. Addition of doxycycline markedly decreases expression of MYCN, with little MYCN expressed in the *MYCN* non-amplified cell lines CHLA255, SK-N-SH, or SH-SY5Y. **(B)** Western blot showing MYCN is overexpressed in SHEP‑21N without doxycycline (-dox), comparable to MYCN expression seen in the *MYCN* amplified cell line NGP. Addition of doxycycline (+dox) to SHEP‑21N turns off expression of MYCN. The *MYCN* nonamplified cell line SH-SY5Y expresses little MYCN. Bottom lane β-actin loading control.

**Supplementary Figure S2**

**
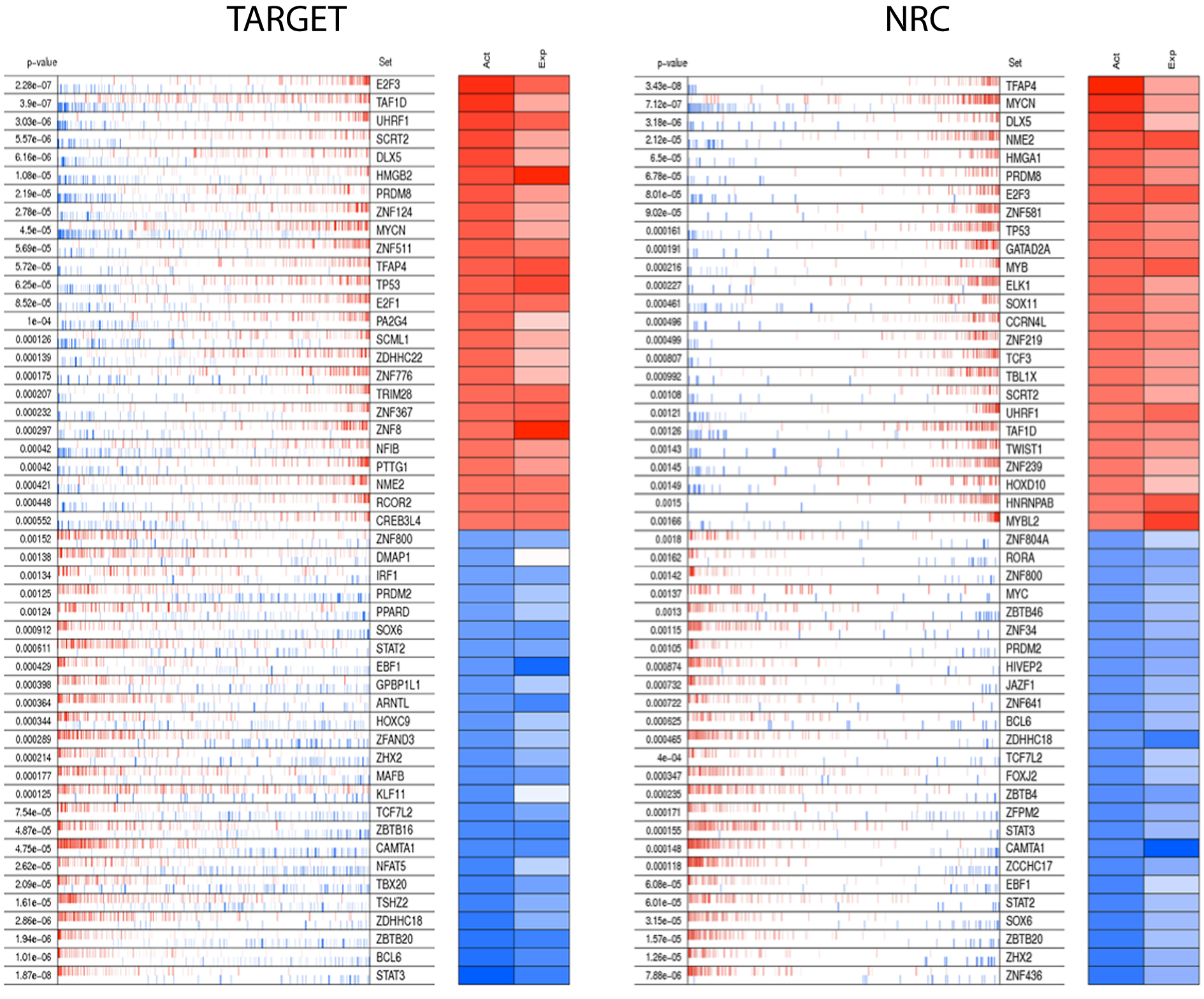
**

**Fig. S2.** **MARINa analysis identifies top 25 master regulators in *MYCN* amplified neuroblastoma in TARGET and NRC datasets**. The map shows distribution of positively (red) and negatively (blue) regulated targets of each MR ranked by differential activity (Act) between *MYCN*-amplified versus stage 1 patient samples. Differential expression (Exp) is shown in the right hand column.

**Supplementary Figure S3**

**
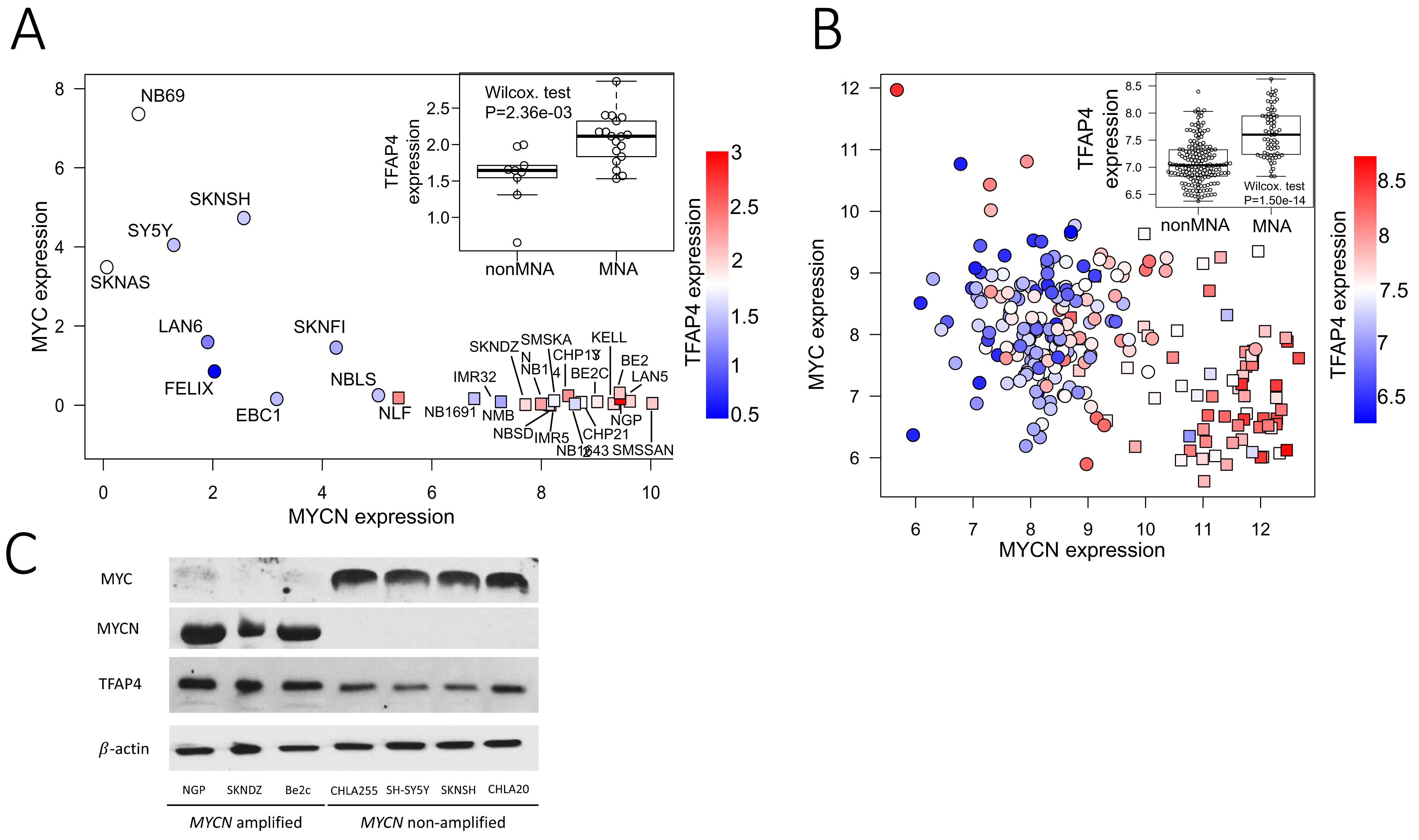
**

**Fig. S3. *TFAP4* expression in neuroblastoma cell lines and patients.** (A) Scatter plot representing MYCN and MYC expression in *MYCN*-amplified 🞎 and *MYCN* non-amplified ⭘ samples in neuroblastoma cell lines. Color intensity represents relative *TFAP4* expression in these cell lines. (B) Scatter plot representing MYCN and MYC expression in *MYCN*-amplified 🞎 and *MYCN* non-amplified ⭘ samples from the TARGET cohort. Color intensity represents relative *TFAP4* expression in each patient. (C) Western blot showing MYC, MYCN and TFAP4 protein level in different neuroblastoma cell lines.

**Supplementary Figure S4**

**
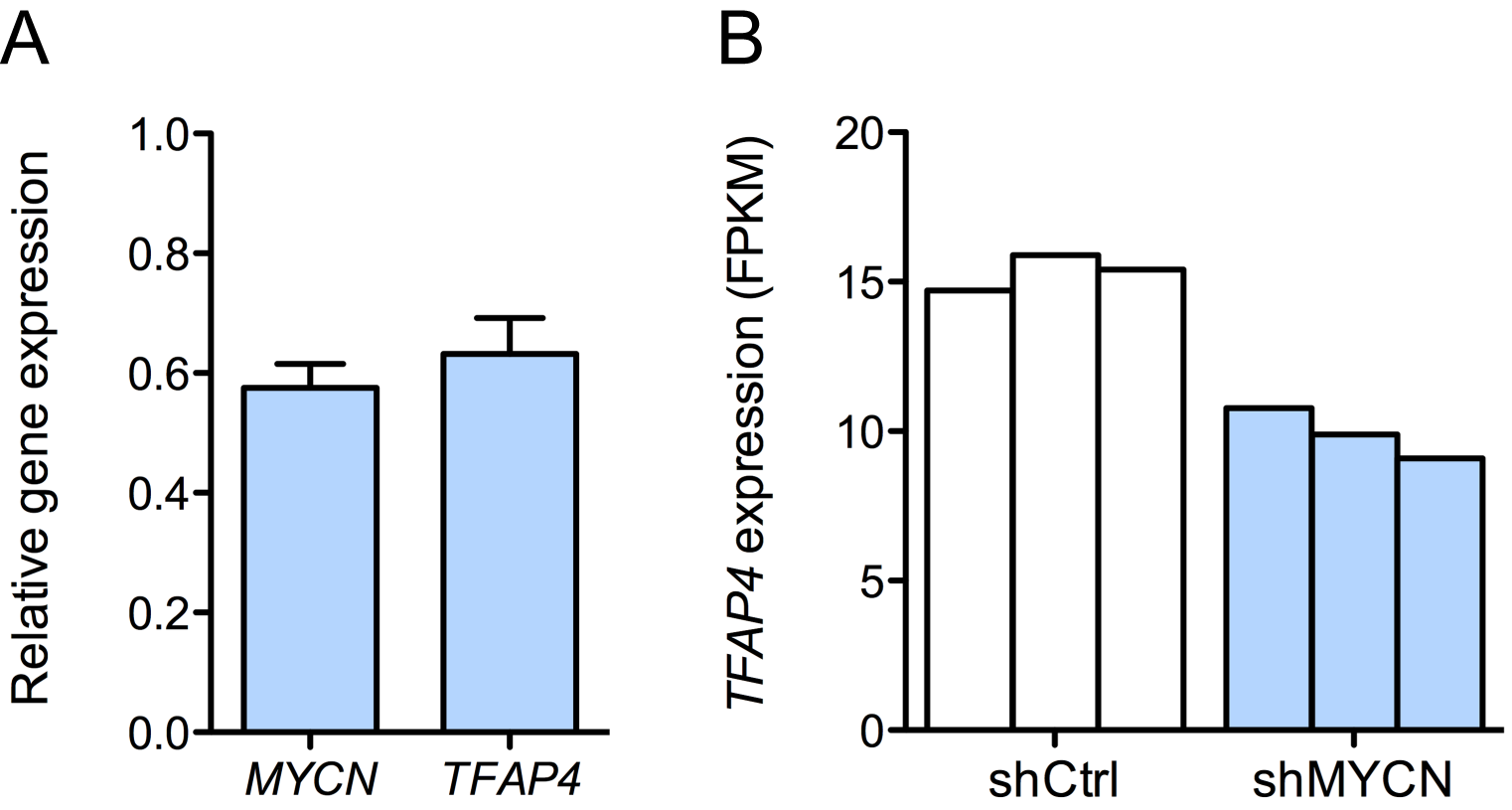
**

**Fig. S4. MYCN regulates the expression of TFAP4.** The *MYCN*-amplified neuroblastoma cell line SK-N-BE(2) was transduced with a MYCN shRNA or control shRNA and RNA extracted 48 hours later. (A) The relative expression of *MYCN* and *TFAP4* was quantified by qPCR, mean +/- S.E.M. (B) In a separate experiment, the transcriptome of SK-N-BE(2) transduced with MYCN shRNA or control shRNA was determined by RNAseq (three replicates per condition). Expression of *TFAP4* expression was significantly decreased in shMYCN, T statistic: -9.74; *P‑*value: 2.09e-05; FDR: 3.02e-04. Fragments Per Kilobase of transcript per Million mapped reads (FPKM)

**Supplementary Figure S5**

**
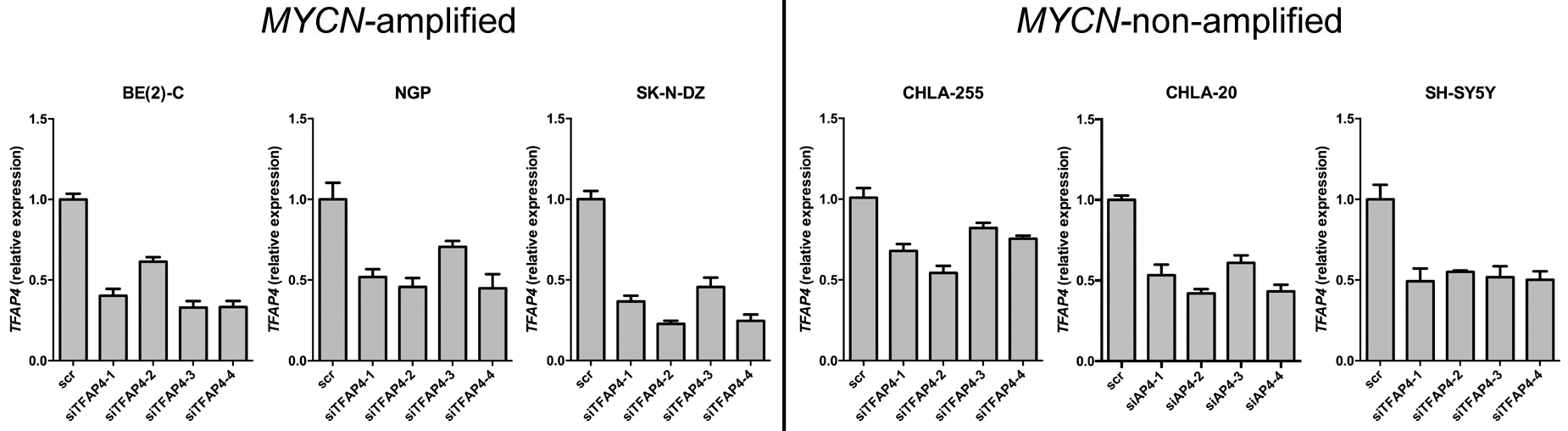
**

**Fig. S5.** **siRNA silencing of TFAP4.** Four different constructs of siRNAs were used to silence TFAP4 and compared to scrambled siRNA. 40 hrs after siRNA transfection expression of *TFAP4* was quantified by RT-PCR.

**Supplementary Figure S6**

**
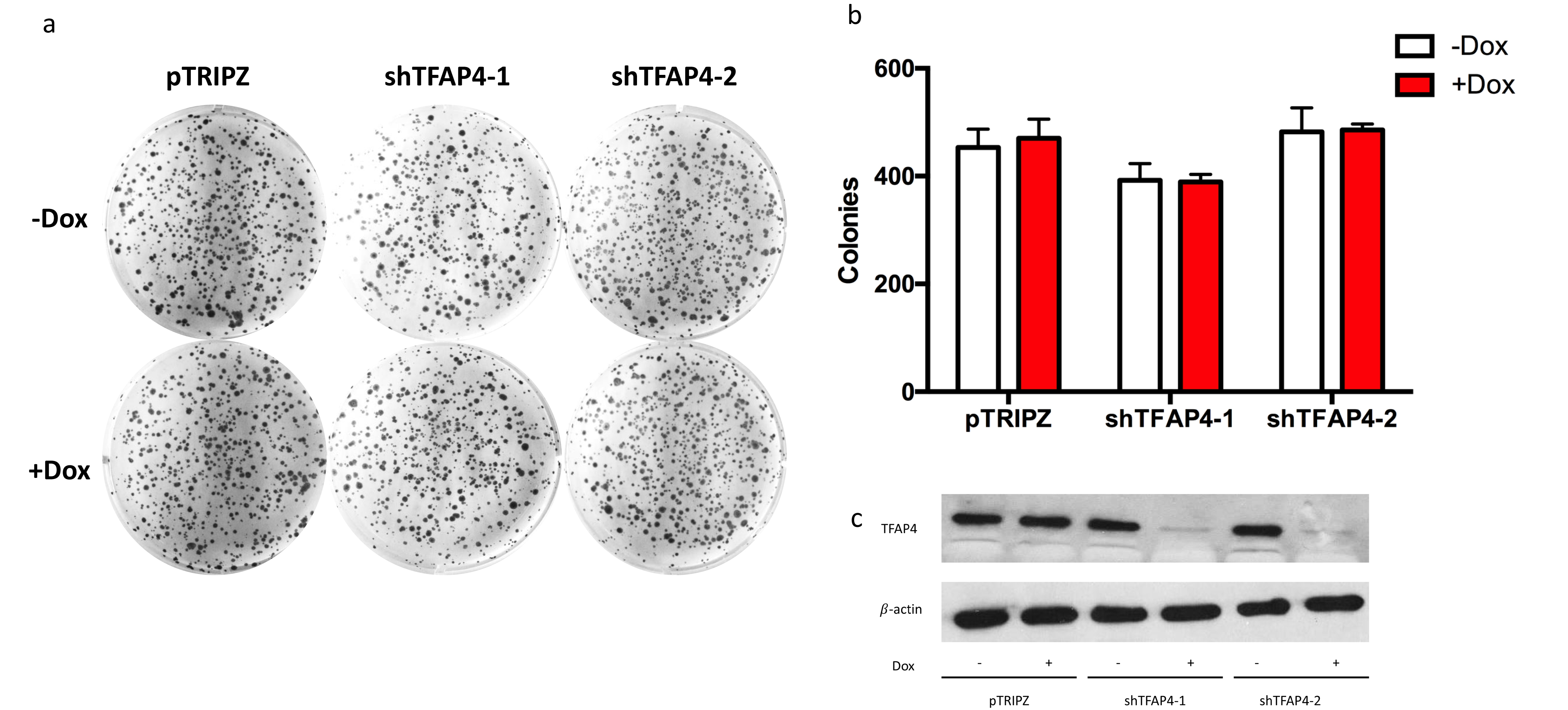
**

**Fig. S6. Silencing of *TFAP4* in CHLA-20 *MYCN* non-amplified cells.** CHLA-20 were infected with two different dox-inducible shRNAs against *TFAP4* as well as the empty vector control pTRIPZ*.* shRNA was induced by 1 μg/ml doxycycline at day 0. Protein lysates were collected at day 3. Silencing of *TFAP4* was confirmed by Western blot.

**Supplementary Figure S7**

**
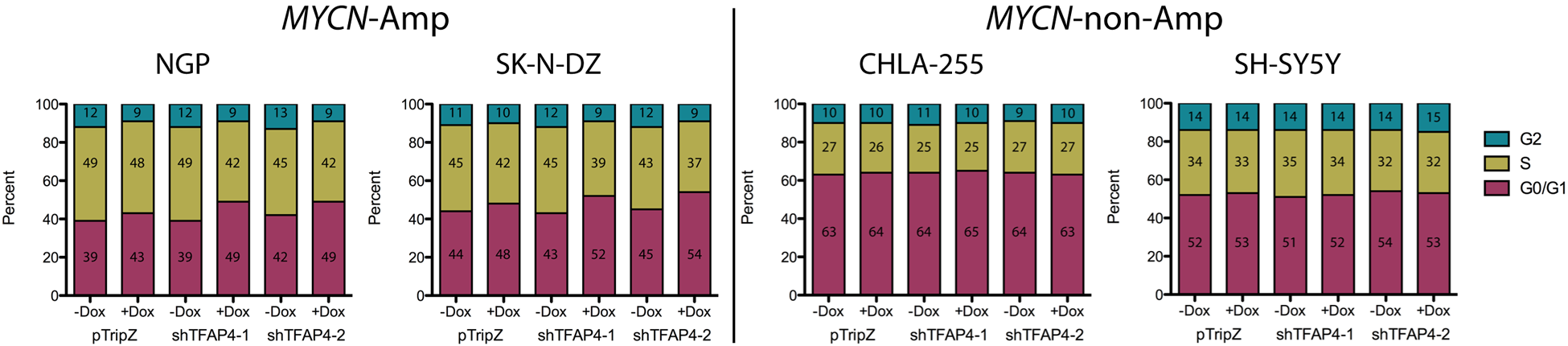
**

**Fig. S7. Silencing of TFAP4 inhibits progression through the cell cycle in *MYCN*-amplified cell lines.** Bar graph of percentage of cells in G0/G1 phase, G2 phase and S phase. Cells were collected three days after doxycycline induction. Experiments were performed in triplicate.

**Supplementary Figure S8**

**
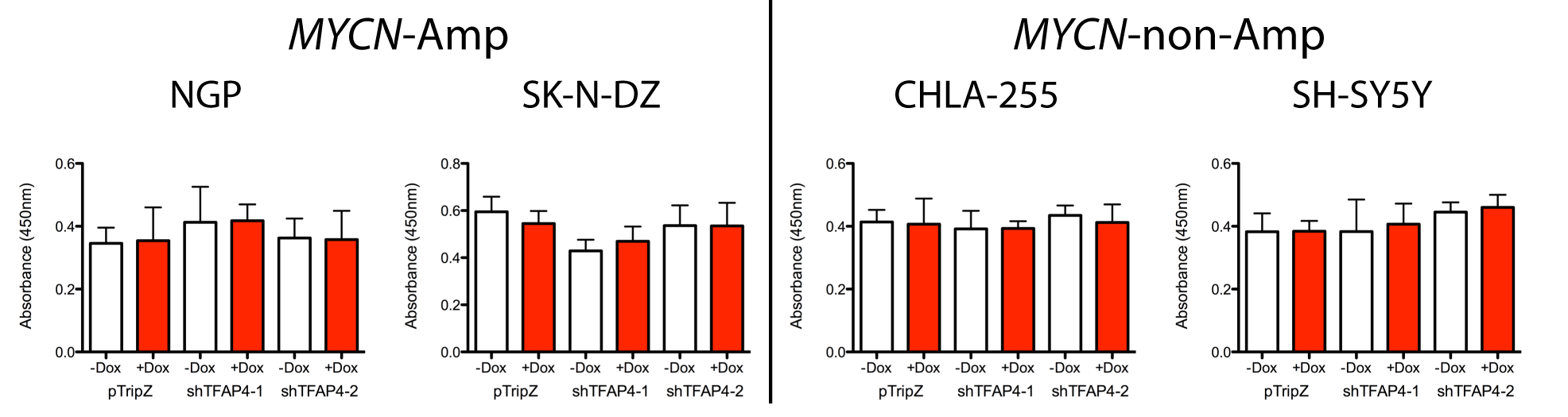
**

**Fig. S8. Silencing of *TFAP4* does not induce apoptosis.** Neuroblastoma cells were plated on 96 wells plate with or without doxycycline. Three days after doxycycline induction, cell apoptosis was measured by TUNEL assay. Experiments were performed in quadruplicates.

**Supplementary Figure S9**

**
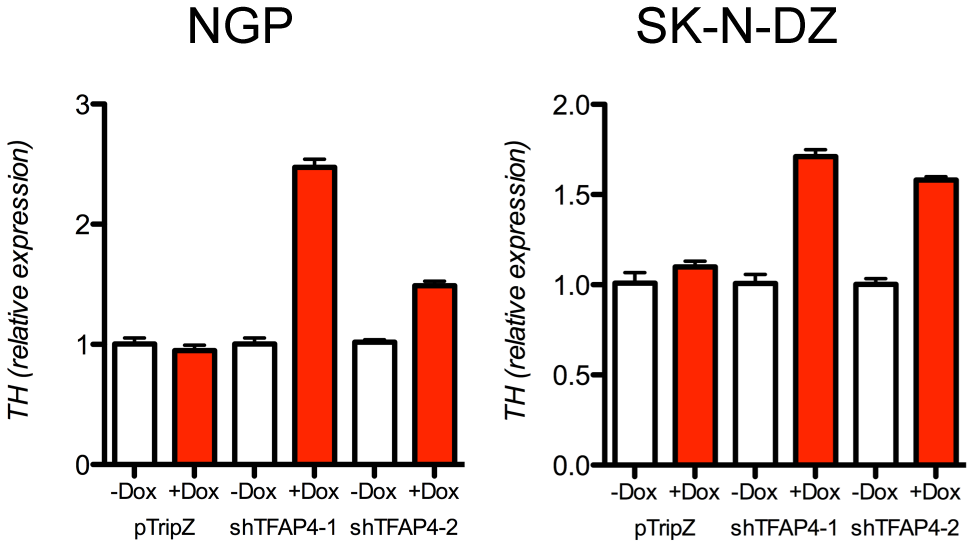
**

**Fig. S9. Silencing of *TFAP4* increases expression of *TH*.** Gene expression for *TH* was measured by quantitative PCR 4 days after induction of shRNA against *TFAP4.*

**Supplementary Figure S10**

**
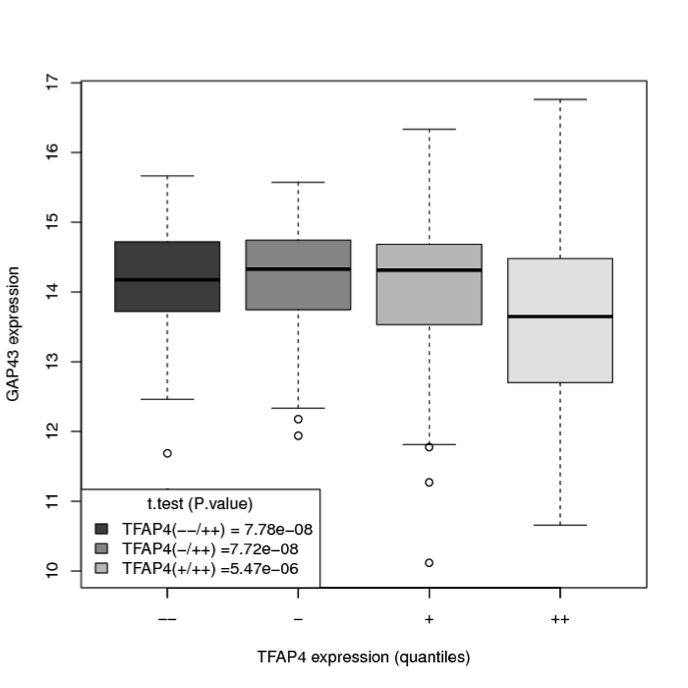
**

**Fig. S10. *TFAP4* is inversely correlated with *GAP43* in human neuroblastoma.** Utilizing the TARGET data set, *TFAP* expression was divided into quartiles and *GAP43* expression plotted. Patients with the highest *TFAP* expression (++) had significantly lower *GAP43* expression then those with lower TFAP expression quartiles (+), (-), (--), with *P*=5.47e-6, *P*=7.72e-8. *P*=7.79e-8, respectively.

**Supplementary Figure S11**

**
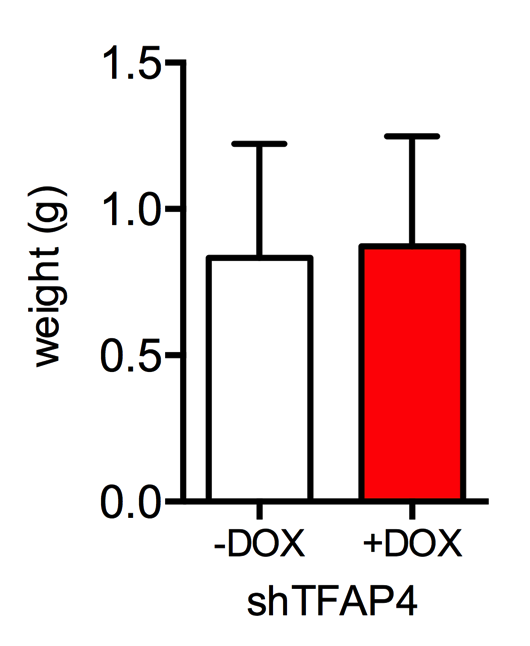
**

**Fig. S11. No difference in tumor weight between the *TFAP4* silenced group (+Dox) compared to the control (-Dox).** Tumor weight was measured at the sacrifice point, when the primary tumor luciferase flux reached 6x10^9^ photons/sec.

**Supplementary Figure S12**


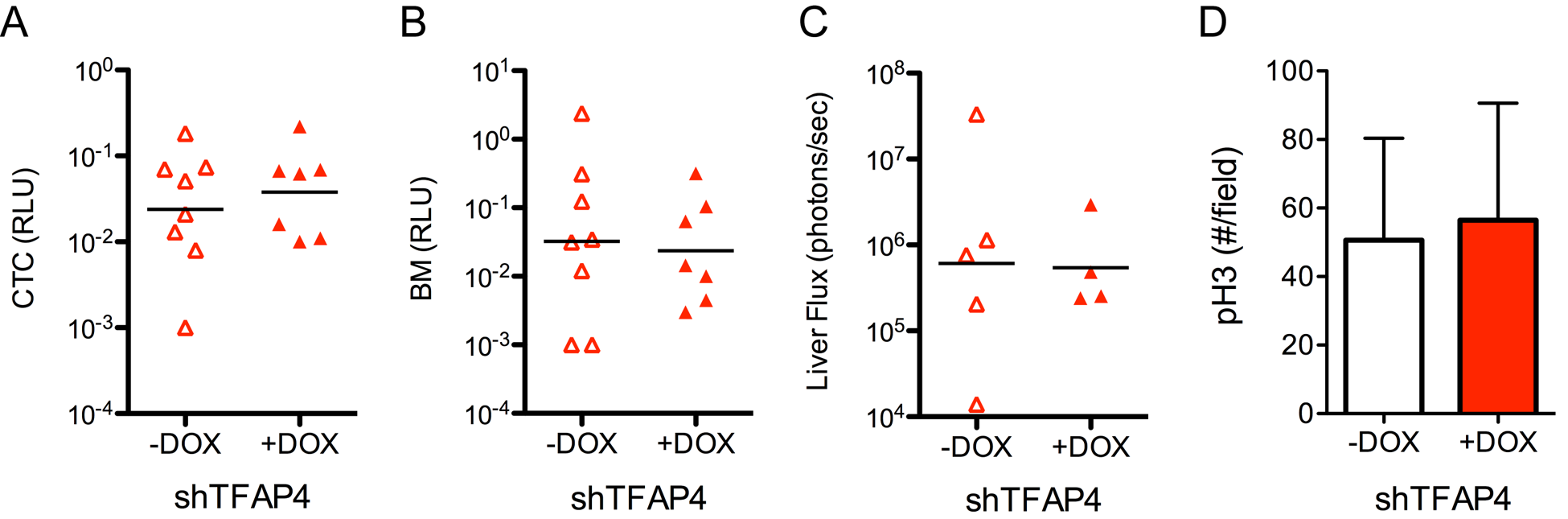


**Fig. S12.** **Silencing TFAP4 does not affect metastasis.** Bioluminescence of luciferase labeled NGP shTFAP cells was quantified in: (A) Peripheral blood to determine circulating tumor cells (CTC); (B) Bone marrow; or in C) Liver; with or without doxycycline.

**Supplementary Figure S13**

**
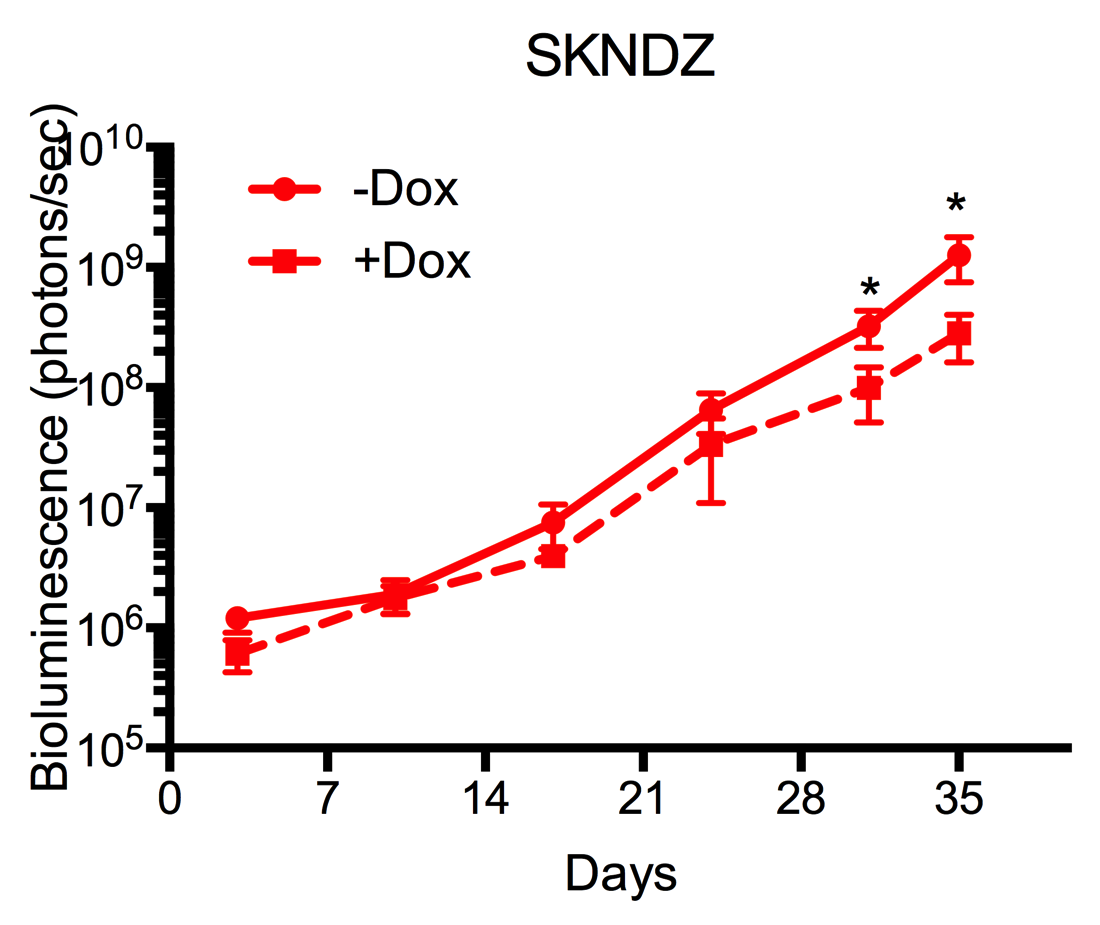
**

**Fig. S13. Silencing TFAP4 inhibits SK-N-DZ tumor growth.** Luciferase activity of the NGP tumor over time. *, *P*<0.05. 10^6^ luciferase labeled SK-N-DZ cells with dox-inducible shTFAP4 were implanted into the kidney of athymic mice. Bioluminescence imaging was taken once a week. Mice were randomized 3 days after tumor implantation with one group of mice given drinking water with doxycycline (+Dox, n=9), and the other group without (-Dox, n=8).

**Supplementary Figure S14**


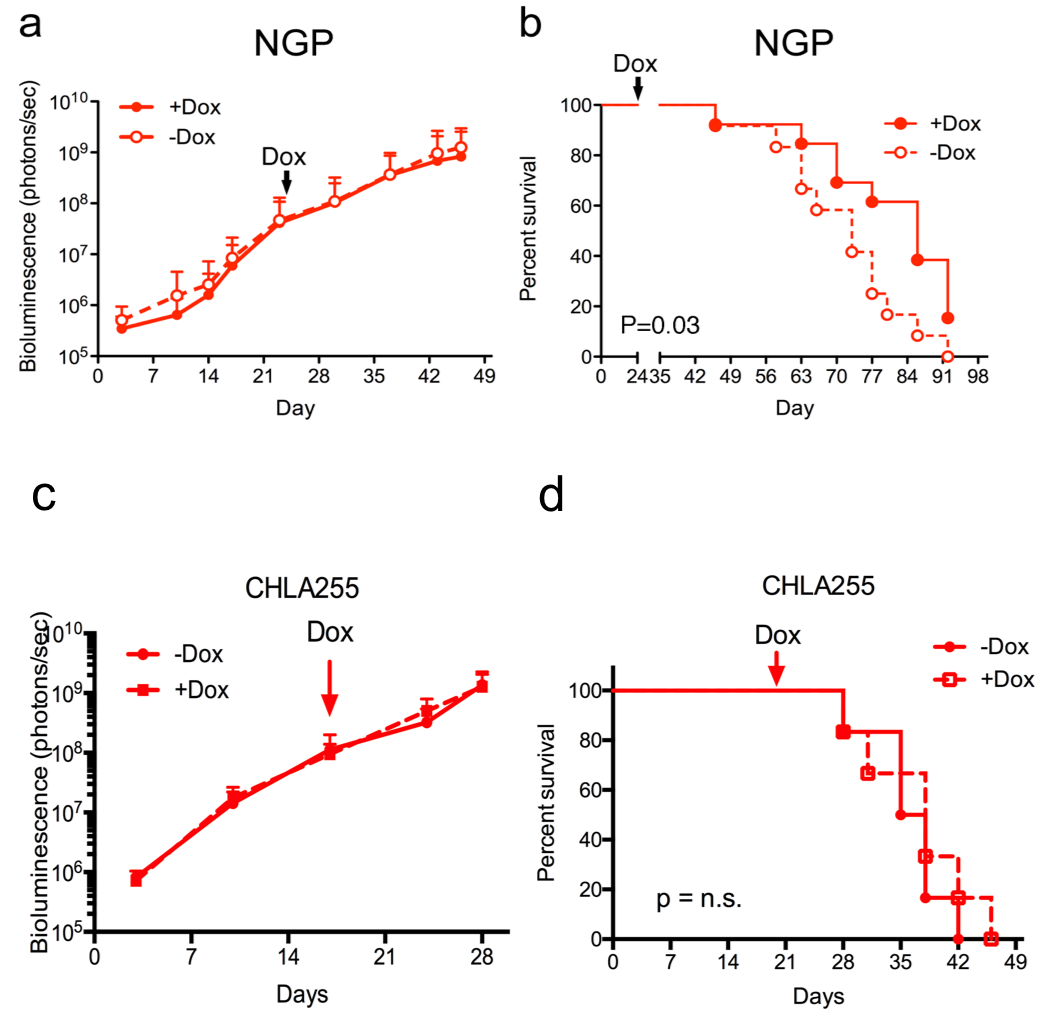


**Fig. S14. Silencing TFAP4 prolongs survival of mice with established MYCN-amplified tumors, but not MYCN-non-amplified tumors.** (A) 10^6^ luciferase labeled NGP cells with dox-inducible shTFAP4 were implanted into the kidney of athymic mice. Bioluminescence imaging was taken once a week. Mice were randomized 24 days after tumor implantation with one group of mice given drinking water with doxycycline (+Dox, n=13), and the other group without (-Dox, n=12). (B) Kaplan-Meier curve of NGP mice with TFAP4 knocked down (+Dox) or control (-Dox). Mice were sacrificed when luciferase activity reached 6x10^9^ photons/sec. Silencing of TFAP4 in NGP tumors significantly prolonged overall survival by 13 days, with median survival 86 vs 73 days, +Dox vs -Dox, respectively P = 0.03. (C) 10^6^ luciferase labeled CHLA-255 cells with dox-inducible shTFAP4 were implanted into the kidney of athymic mice. Bioluminescence imaging was taken once a week. Mice were randomized 17 days after tumor implantation with one group of mice given drinking water with doxycycline (+Dox, n=6), and the other group without (-Dox, n=6). (D) Kaplan-Meier curve of CHLA-255 mice with TFAP4 knocked down (+Dox) or control (-Dox). Mice were sacrificed when luciferase activity reached 6x10^9^ photons/sec.

**Supplementary Figure S15**


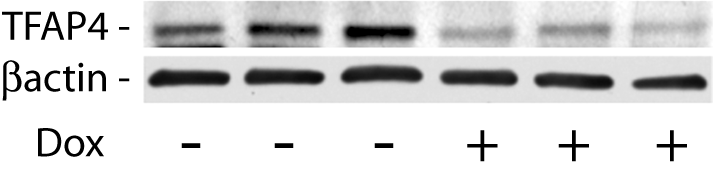


**Fig. S15**. **Silencing of TFAP4 in CHLA-255 xenograft tumors.** Treatment with doxycycline (+) decreased the expression of TFAP4 in xenograft tumors of the *MYCN* non-amplified cell line CHLA-255 shTFAP4.

**Supplementary Figure S16**

**A**

**B**

**Fig. S16. GSEA pathway enrichment analysis.** Gene set enrichment analysis (GSEA) was performed in order to calculate differentially active pathways and gene sets. Gene set collections where obtained from MSigDB database. MSigDB C2 curated pathways (A) up-regulated upon TFAP4 knockdown (p<0.01), and (B) down-regulated upon TFAP4 knockdown (p<0.01).
